# Unsolved Mysteries in NLR Biology

**DOI:** 10.3389/fimmu.2013.00285

**Published:** 2013-09-17

**Authors:** Christopher Lupfer, Thirumala-Devi Kanneganti

**Affiliations:** ^1^Department of Immunology, St. Jude Children’s Research Hospital, Memphis, TN, USA

**Keywords:** inflammasomes, NOD-like receptors, DAMPs, PAMPs, innate immunity, caspase-1, embryonic development

## Abstract

NOD-like receptors (NLRs) are a class of cytoplasmic pattern-recognition receptors. Although most NLRs play some role in immunity, their functions range from regulating antigen presentation (NLRC5, CIITA) to pathogen/damage sensing (NLRP1, NLRP3, NLRC1/2, NLRC4) to suppression or modulation of inflammation (NLRC3, NLRP6, NLRP12, NLRX1). However, NLRP2, NLRP5, and NLRP7 are also involved in non-immune pathways such as embryonic development. In this review, we highlight some of the least well-understood aspects of NLRs, including the mechanisms by which they sense pathogens or damage. NLRP3 recognizes a diverse range of stimuli and numerous publications have presented potential unifying models for NLRP3 activation, but no single mechanism proposed thus far appears to account for all possible NLRP3 activators. Additionally, NLRC3, NLRP6, and NLRP12 inhibit NF-κB activation, but whether direct ligand sensing is a requirement for this function is not known. Herein, we review the various mechanisms of sensing and activation proposed for NLRP3 and other inflammasome activators. We also discuss the role of NLRC3, NLRP6, NLRP12, and NLRX1 as inhibitors and how they are activated and function in their roles to limit inflammation. Finally, we present an overview of the emerging roles that NLRP2, NLRP5, and NLRP7 play during embryonic development and postulate on the potential pathways involved.

## Introduction

Innate immunity is initiated by germline-encoded pattern-recognition receptors (PRRs). Among these, the nucleotide oligomerization and binding domain (NOD)-like receptors (NLRs) comprise a large receptor family of more than 20 members (1–4). Only about half of the NLRs have been characterized in any detail. However, it is well documented that NLRs play a critical role in protection against infectious diseases, including bacteria (5, 6), viruses (7, 8), fungi (9, 10), protists (11, 12), and helminthes (13). Of the NLRs which have been studied, most of them fall into one of four categories: (1) Inflammasome activators, (2) Activators of Nuclear Factor-κB (NF-κB) and mitogen activated protein kinase (MAPK), (3) Inhibitors of inflammatory signaling, (4) and trans-activators of MHC expression. However, several NLRs have definite roles in embryogenesis, uterine implantation, and fetal development (14). Intriguingly, some NLRs appear to play multiple roles within inflammation or development. This suggests alternative functions for some NLRs in different cell types or multiple activation mechanisms with separate downstream effects for other NLRs.

One set of NLRs that regulates NF-κB and MAPK are NLRC1 (NOD1 or CARD4) and NLRC2 (NOD2 or CARD15). NLRC1 recognizes iE-DAP, a subunit of peptidoglycan found in some bacterial cell walls (5, 15). NLRC2 recognizes MDP, another peptidoglycan fragment (16–19). NLRC1 and NLRC2 then act through the adaptor RIPK2 to activate NF-κB and MAPK signaling (20–22). However, there are now multiple reports that demonstrate NLRC2 can respond to cytosolic RNA during viral infection (23–25). Although viral RNA also induces an interaction between NLRC2 and RIPK2, this appears to regulate autophagy mechanisms, instead of NF-κB, and subsequently represses inflammasome activation and prevents immunopathology (25). Furthermore, viral RNA mediated activation causes NLRC2 to interact with the antiviral adaptor protein MAVS. This interaction was shown to be essential for the production of IFN-β during viral infection and for suppressing virus replication (24). Additionally, NLRC2 regulates other antiviral pathways like 2′-5′ oligoadenylate synthease (OAS2), which activates RNAse L and degrades viral RNA, thus potentiating antiviral signaling (23). It is not clear how NLRC2 would bind to both MDP and RNA, but one possibility is that additional upstream adaptor proteins, which have yet to be discovered, actually provide specificity.

In the case of NLRC4, it is activated by bacterial flagellin (26, 27) or the rod complex of bacterial type III secretion systems (T3SS) (28). Once activated, NLRC4 forms a multimeric complex, known as the inflammasome, with the adaptor ASC and caspase-1 (26, 27). Inflammasome formation results in a proinflammatory cell death termed pyroptosis (29) and the release of IL-1β and IL-18 (30–34). Recently, the ability of NLRC4 to recognize flagellin and T3SS components was tagged to an association between NLRC4 and another class of NOD proteins known as NAIPs. NAIP5 and NAIP6 in the mouse recognize flagellin and NAIP2 recognizes T3SS rod complexes respectively and then activate NLRC4 (35–37). Furthermore, NAIP1 in mice activates NLRC4 in response to the needle protein of some T3SS (38). In human cells, only one NAIP exists, and this recognizes the needle protein of T3SS similar to mouse NAIP1 (38). These results demonstrate that one mechanism for the recognition of multiple ligands by NLRs is the presence of upstream adaptor proteins like NAIPs.

Distinct NLRs recognize microbial or viral components such as peptidoglycan, flagellin, or viral RNA. These pathogen specific molecules are known as pathogen-associated molecular patterns (PAMPs). Alternatively, some NLRs, like NLRP3, detect damage associated molecular patterns (DAMPs). DAMPs consist of byproducts of pathogen invasion or sterile cellular damage such as uric acid crystals, reactive oxygen species (ROS), or extracellular ATP release (39–42). Sensing of DAMPs by NLRP3 is not only critical for detection and clearance of pathogens but also for protection and repair of tissues during inflammation (43, 44). NLRP3 is one of the most ubiquitously important NLRs. Once activated, NLRP3 also forms an inflammasome with the adaptor ASC and caspase-1 (45, 46). NLRP3 responds to an incredibly broad range of pathogens making it unlikely that it senses PAMPs directly. Many lines of evidence support a role for NLRP3 in DAMP sensing, where damage to the host results in the release of certain danger signals not present under homeostatic conditions. ROS, potassium efflux, and release of proteases from endosomes have all been reported to activate NLRP3 (41, 42, 47–51). Although much is know about the range of stimuli that can activate NLRP3, much research remains to be done to understand how NLRP3 becomes activated.

The ability of NLRP3 to respond to multiple PAMPs or DAMPs from such a broad range of pathogens strongly indicates the presence of upstream adaptors, as is the case for NLRC4, or common danger signals which funnel into one pathway. In the case of NLRC2, the different signaling pathways activated by MDP or viral RNA would suggest that different modes of NLRC2 activation lead to different protein conformations or other alterations in NLRC2 activity. This is subsequently responsible for activation of NF-κB, autophagy, or antiviral signaling. Indeed, these are some of the great-unsolved mysteries of NLR biology. In addition, other NLRs, like NLRC3, NLRP6, NLRP12, and NLRX1, play inhibitory roles during inflammation. Yet how these proteins are activated or perform their inhibitory functions is not well understood. Finally, there are numerous NLRs for which there are different reports indicating a multiplicity of potential functions. In this review, we discuss several of these unsolved mysteries and potential future directions in the field of NLR biology.

## Activation Mechanisms of NLRP3

**Figure 1:**
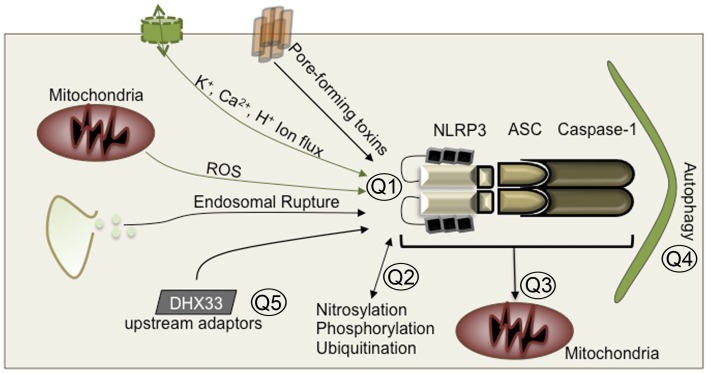
**Unsolved mysteries in NLRP3 biology**. Q1: Is there a common DAMP that activates NLRP3? Do DAMPs directly activate NLRP3? Do DAMPs induce structural rearrangement of NLRP3? Q2: How do post-translational modifications regulate activation on a structural level? Q3: Is mitochondrial localization essential for NLRP3 inflammasome formation? Q4: How does autophagy inhibit NLRP3? Does autophagy directly engulf NLRP3 inflammasomes? Does it engulf damaged mitochondria where NLRP3 is localized? Does autophagy merely remove the source of DAMPs? Q5: Are there additional upstream sensors or adaptors that facilitate NLRP3 activation?

NLRP3 was initially described as an activator of caspase-1 in 2002 and was subsequently associated with autoinflammatory periodic fevers like Muckle–Wells syndrome or bacterial infection (45, 46). Since then, there have been many proposed mechanism for how NLRP3 is activated. There is no evidence that NLRP3 interacts directly with any PAMP. Although NLRP3 is activated in response to bacteria and viral RNA (7, 52), lipopolysaccharide, and MDP (53), most PAMPs appear to only be required for the transcriptional up-regulation of NLRP3 and pro-IL-1β (54). Once NLRP3 is upregulated, a second signal, generally a DAMP or a pore forming toxin like nigericin, is required for NLRP3 to interact with ASC and caspase-1 to form an active inflammasome. This second signal is frequently associated with the production of ROS or endosomal rupture (41, 42, 49, 50). Changes in intracellular and extracellular calcium (55–58) and potassium efflux (47, 48, 51) have also been proposed to activate NLRP3, as have changes in cytosolic or extracellular pH (59, 60). This dichotomy of signals for priming and activation is required for NLRP3 inflammasome formation. The big question that remains is how ROS, ion flux, or other DAMPs regulate NLRP3 (Figure 1). One possibility is that the structure of NLRP3 is sensitive to changes in ion concentrations, and exposure of the pyrin effector domain occurs when ion concentrations deviate from their homeostatic state (48, 51). Alternatively, protein sensors of cellular redox or ion sensors could regulate NLRP3 activation following their own activation. Studies into the structure of NLRP3 and the effects of different ions on the ATPase activity, pyrin effector domain exposure, and ASC binding affinity of NLRP3 would greatly increase our understanding of how NLRP3 is activated.

Proteomic studies directed at understanding the NLRP3 interactome using different activators of NLRP3 may also provide further insight into potential upstream regulators such as NAIPs or ion/ROS sensors. A recent paper by Mitoma et al. (61) found in human macrophages that NLRP3 is activated in responses to double stranded RNA through an interaction with the RNA helicase DHX33 (61). Protein kinase R (PKR) is an RNA dependent kinase involved in antiviral defenses. Activation of NLRP3 was also proposed to be dependent on PKR, although phosphorylation of NLRP3 was not required (62). However, another group attempted to examine the role of PKR mediated activation of NLRP3 but found no role for PKR (63). Finally, the adaptor protein MAVS, which is required for antiviral signaling downstream of the RNA helicases RIG-I and MDA5, has been shown to interact with NLRP3 and regulate its activation and localization to the mitochondria (64). Although this was shown in the context of RNA transfection, LPS + ATP treatment or nigericin, how LPS or nigericin could activate MAVS remains to be investigated further. In all, there is a significant body of research that would indicate the presence of upstream PRR that tie into the NLRP3 pathway (Figure 1).

Although multiple upstream sensors may regulate NLRP3, it is possible that NLRP3 interacting partners regulate its activation through the addition or removal of post-translational modifications. Post-translational modification of NLRs has been reported to regulate their activation. For example, phosphorylation of NLRC4 by PKCδ regulates its activation during *Salmonella typhimurium* infection in macrophages (65). In the case of NLRP3, nitric oxide produced during chronic inflammation *in vivo* during *Mycobacterium tuberculosis* infection results in nitrosylation of NLRP3 and inhibition of inflammasome activation (66). Similarly, the addition of NO donor compounds to macrophages or induction of NO by IFN-γ treatment inhibited NLRP3 activation (66, 67). The role of NO for NLRP3 inhibition during LPS-induced sepsis in mice has also been reported (68). Therefore, proteins that can regulate the nitrosylation status of NLRP3 may be able to regulate its activation. Ubiquitination and deubiquitination were also found to regulate NLRP3 activation (69, 70). Thus far, deubiquitination by the BRCC3 deubiquitinase is the only post-translational modification that is reported to activate NLRP3 (70). It is clear that post-translational modifications can affect NLRP3 activation, although how ubiquitination, or nitrosylation affect the function of NLRP3 needs further biochemical examination (Figure 1).

The cellular autophagy pathway, which is required for recycling damaged organelles and proteins, has been reported to inhibit NLRP3 activation. Ubiquitinated inflammasomes are degraded through the autophagy pathway (71). This report, in combination with those above, may indicate that deubiquitination of NLRP3 prevents autophagic degradation and allows for inflammasome formation. Alternatively, the removal of damaged mitochondria, which produce NLRP3 activators like ROS or release of mitochondrial DNA into the cytosol, constitutes another mechanism by which autophagy regulates NLRP3 activation (25, 72, 73). It is also possible that autophagosomal degradation of damaged mitochondria simultaneously removes inflammasomes. Several recent publications demonstrate that NLRP3 inflammasome formation is dependent on localization to the mitochondria (64, 74). However, another report demonstrated that inflammasome activation was not associated with any organelle but occurred in the cytosol (75). Why there are conflicting reports regarding the mechanisms that activate NLRP3 are unclear. However, in the case of cellular localization, differences in fixation or staining methodologies may result in aggregation of inflammasomes with mitochondria or their disassociation, respectively. In all, mitochondria appear to play a role in the regulation of NLRP3 inflammasome activation, but whether they serve as an activation platform, a source of stimuli, or both requires further investigation (Figure 1).

To more fully understand NLRP3 regulation, the interactome of NLRP3 including kinases and ubiquitin ligases still need to be discovered and the regulation of post-translational pathways examined. Clearly there is need for a concerted effort from biochemists, molecular and structural biologists, and immunologists to collaborate on these issues. As NLRP3 is associated with numerous autoinflammatory and autoimmune diseases, understanding how NLRP3 is regulated will be necessary for understanding and potentially preventing disease development, as well as for the design of inhibitors which are useful under specific inflammatory conditions.

## Regulation of Inhibitory NLRs

**Figure 2:**
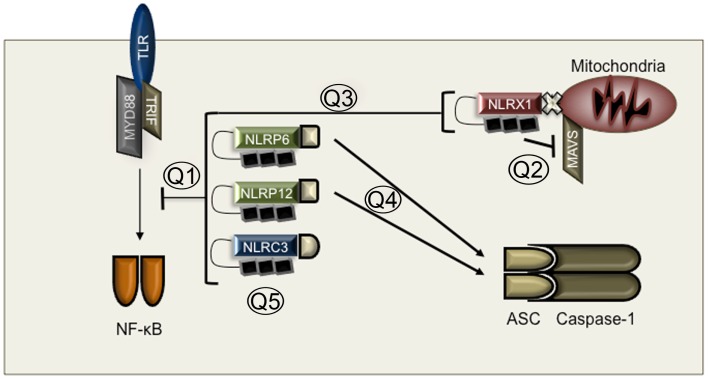
**Mechanisms of inhibitory NLRs**. Q1: How do inhibitory NLRs function? Is PAMP recognition required for inhibitory NLR function? Is NLR expression sufficient for inhibitory function? Q2: Is NLRX1 an inhibitor of MAVS or a modulator of mitochondrial ROS? Q3: How does NLRX1 inhibit NF-κB if it is localized to the mitochondria? Q4: Do NLRP6 and NLRP12 regulate inflammasome activation and how? Does gut flora play a role in inflammasome activation in the absence of NLRP6 and NLRP12. Q5: Why are there so many inhibitory NLRs? Do inhibitory NLRs play redundant or context specific roles?

Intriguingly, all inhibitory NLRs studied thus far have been found to inhibit NF-κB activation. NLRP12 was examined during colon inflammation and colon tumorigenesis and found to negatively regulate NF-κB down stream of Toll-like receptors (TLRs) (76, 77) or to regulate the alternative NF-κB pathway downstream of TNF family receptors (76, 78). NLRP12 appears to interact with NF-κB–inducing kinase (NIK), interleukin-1 receptor-associated kinase 1 (IRAK1), and TNF receptor-associated factor 3 (TRAF3), which are known mediators of NF-κB signaling (78, 79). These interactions appear to regulate the phosphorylation of IRAK1 and the degradation of NIK, thus resulting in inhibition of the alternative NF-κB pathway. However, the mechanism by which NLRP12 inhibits TLR mediated activation of the classical NF-κB pathway is not known (Figure 2).

Currently, it is unclear how the inhibitory function of NLRP12 is regulated (Figure 2). ATP binding appears to be a requirement for activation (79) but the mechanism by which NLRP12 structural rearrangement occurs to permit ATP binding has not been examined. NLRP12 expression increases following NF-κB activation (80). It is also apparent that NLRP12 interacts with other proteins which regulate its function, including HSP90, which stabilizes NLRP12 and prevents its proteasomal degradation (81). Whether expression alone is sufficient for its inhibitory function, or if NLRP12 is regulated by post-translational modifications of some kind is unclear.

Recently, *Nlrc3*-deficient mice were generated and inflammation examined in response to LPS treatment (82). Sub-lethal LPS administration resulted in increased IL-6, increased macrophage numbers and increased hypothermia in *Nlrc3*^−/−^ mice. Examination of *Nlrc3*^−/−^ macrophages showed that there was enhanced NF-κB activation down stream of TLR signaling (82). Mechanistically, NLRC3 appears to regulate TRAF6 activation by modulating its K^63^-linked ubiquitination and stability. Once again, the mechanisms that regulate NLRC3 activation remain to be examined (Figure 2).

Similar to NLRC3 and NLRP12, NLRP6 also inhibits NF-κB activation down stream of TLR signaling. NLRP6 was shown to suppress NF-κB activation during *Listeria monocytogenes* and *Salmonella typhimurium* infection, and in the absence of NLRP6, bacteria were cleared more rapidly (83). In other studies, *Nlrp6* deficiency predisposes mice to increased inflammation in models of colitis and to increased tumorigenesis in colon cancer models (84, 85). However, the mechanisms proposed for susceptibility to colitis and tumorigenesis are reportedly due to NLRP6 mediated inflammasome activation. *Nlrp6*^−/−^ mice have reduced IL-18 in the colon in these models (84, 85). It should be noted, though, that no biochemical or molecular evidence for an NLRP6 inflammasome has been presented to date. It is therefore possible that NLRP6 regulates inflammasome activation indirectly. In fact, there are significant differences in the gut microbiota in *Nlrp6*^−/−^ mice in the above models, which could result in altered inflammasome activation (Figure 2).

NLRP12 has also been proposed to form an inflammasome. During *Yersinia pestis* infection, NLRP12 is reported to recognize acylated lipid A and *Nlrp12*^−/−^ mice were more susceptible to infection and had reduced IL-18 levels (86). In humans, NLRP12 polymorphisms are associated with inflammasome activation during periodic fever syndromes (87, 88). It is possible that both NLRP12 and NLRP6 have regulatory roles during NF-κB activation as well as in inflammasome formation. To verify these functions, however, much needs to be done on the molecular and biochemical level to determine the mechanisms by which these proteins activate the inflammasome and what stimuli activate them to form an inflammasome verses inhibit NF-κB activation. Finally, as discussed above, several NLR deficient mouse strains have been found to harbor altered gut microbiota compared to WT controls. The ability of the microbiome to regulate immunity is clear, but the exact effects of these changes are still not well-understood. Especially during models of colon inflammation, differences in gut flora between mice may be an essential factor in the phenotypes observed. The use of germ free or gnotobiotic mice for studying the roles of NLRs in general, but NLRP12 and NLRP6 in particular, may help resolve their functions as immune activators or repressors and the mechanisms by which they perform these functions.

The last NLR with a proposed inhibitory function is NLRX1. NLRX1 was originally reported to inhibit antiviral signaling through inhibition of the adaptor MAVS (8, 89). Subsequently, NLRX1 was shown to inhibit TLR mediated activation of NF-κB (90). How NLRX1 inhibits NF-κB is not clear though, as NLRX1 is localized to the mitochondria. Furthermore, the role of NLRX1 as an inhibitor is debated. Several groups have found no role for NLRX1 in regulating MAVS but have instead reported NLRX1 as a modulator of mitochondrial ROS (91–93). Recently, the crystal structure of NLRX1 was solved along with biochemical evidence for the binding of NLRX1 to the viral RNA mimic poly(I:C) (94). Although this finding would support a role for NLRX1 in antiviral signaling, the exact function of NLRX1 will require further examination (Figure 2).

As discussed above, the *in vivo* importance of inhibitory NLRs has been demonstrated in various models of inflammation. However, why there are so many NLRs that inhibit NF-κB signaling is a conundrum. If expression of these inhibitory NLRs alone were sufficient to suppress NF-κB activation, then why would there need to be four. One possibility is that they function as a whole to modulate NF-κB activation appropriately. Another possibility is that they are activated only in response to certain infections or stimuli. However, treatment with LPS or poly(I:C) both resulted in increased NF-κB activation in *Nlrp12*^−/−^ macrophages (77), suggesting that ligand recognition is not required for its function. Understanding the individual and combined roles of NLRC3, NLRP6, NLRP12, and NLRX1 during specific infections or models of inflammation will be important as this field moves forward (Figure 2).

## NLRs as Double Agents

As discussed in the last section, NLRP12 and NLRP6 have roles in inhibiting inflammation by modulating NF-κB activation (77–79, 83). In addition, both of these NLRs are reported to regulate inflammasome activation (84–86). As discussed in the introduction, NLRC2 is able to respond to both MDP and viral RNA and activates distinct pathways including NF-κB, autophagy, or antiviral signaling (Table 1). All of these pathways are important for inflammation and immunity. However, NLRs are also implicated in numerous non-inflammatory roles. NLRC1 and NLRC2 have been shown to regulate the differentiation of human umbilical cord blood-derived mesenchymal stem cells (MSC). Although NLRC1 and NLRC2 had no effect on MSC proliferation, they enhanced their differentiation into chondrocytes and osteocytes and inhibited adipocyte formation *in vitro* (95). The ability of NLRC1 and NLRC2 to regulate MSC differentiation was associated with increased ERK1/2 MAPK signaling; a known function of these NLRs (Table 1). The ability of NLRs to affect MSC may play an important part of wound healing and the resolution of inflammation. In fact, NLRP3 was found to play an important function in tissue repair in the lung during influenza A virus infection, although this was likely due to impaired recruitment of macrophages or other cells necessary for wound repair and healing (43).

**Table 1:** **Functionally distinct roles of NLRs in biology**.

NLR	Dual roles	Reference
NLRP12^a^	NF-κB inhibition, caspase-1 activation	Williams et al. (76), Arthur et al. (81), Ye et al. (79), Jeru et al. (87), Jeru et al. (88), Zaki et al. (77), Allen et al. (78), Vladimer et al. (86), Chattoraj et al. (80)
NLRP6^a^	NF-κB inhibition, caspase-1 activation	Chen et al. (84), Elinav et al. (85), Anand et al. (83)
NLRC2^b^	NF-κB and MAPK activation, type-I IFN production, autophagy, MSC differentiation	Bertin et al. (20), Girardin et al. (21), Park et al. (22), Dugan et al. (23), Sabbah et al. (24), Kim et al. (95), Lupfer et al. (25)
NLRP2^c^	Embryonic development, caspase-1 activation	Bruey et al. (96), Fontalba et al. (97), Meyer et al. (99), Peng et al. (101), Huang et al. (100), Minkiewicz et al. (98)
NLRP7^c^	Embryonic development, caspase-1 activation	Murdoch et al. (103), Messaed et al. (104), Khare et al. (102), Huang et al. (100), Ulker et al. (105)

The NLRs listed in this table have been implicated in multiple functional roles. However, the mechanisms by which they perform these distinct roles have not been elucidated. ^a^It is unclear how NLRP6 and NLRP12 function under some inflammatory conditions as inhibitors of NF-κB but under other conditions can serve as inflammasome activators. ^b^NLRC2 responds to a variety of PAMPs including MDP and viral RNA, but the downstream signaling pathways triggered by NLRC2 are distinct for specific PAMPs suggesting alterative activation mechanisms. ^c^Finally, NLRP2 and NLRP7 may serve as inflammasome regulators, but whether their functions in embryonic development are tied to inflammasome activation or are separate functions is unclear.

The role of NLRs in tissue repair or MSC differentiation may be a logical progression following inflammation but several additional NLRs have been reported to regulate seemingly disparate functions. NLRP2 is reported to inhibit NF-κB activation (96, 97) and to enhance caspase-1 activation (96). In addition, siRNA mediated knockdown of NLRP2 in primary human astrocytes was recently reported to impair inflammasome activation (98). How NLRP2 affects inflammasome activation is not entirely clear, as knockdown of NLRP2 resulted in decreased caspase-1 expression as well. Furthermore, the stimulus used for NLRP2 activation was the NLRP3 activator extracellular ATP (98). These findings might indicate that NLRP2 regulates the expression of key NLRP3 inflammasome components as opposed to a novel NLRP2 specific inflammasome. In addition to the role for NLRP2 in inflammasome activation and inhibition of NF-κB signaling, NLRP2 has a definite role in embryonic development (Table 1). A truncation mutation of NLRP2 was found in association with Beckwith–Wiedemann Syndrome (BWS) (99). The NLRP2 mutation resulted in developmental defects that stemmed from altered DNA methylation and gene expression initially present in the maternal oocyte (maternal imprinting) and perpetuated in the fertilized embryo and developing fetus (99). Another study found some association between NLRP2 and recurrent miscarriages (100). Finally, siRNA knockdown of NLRP2 in murine oocytes or embryos leads to nearly complete developmental arrest (101).

Other NLRs have also been proposed to regulate inflammasome activation and development. NLRP7 regulates inflammasome activation in response to acylated lipopeptides like FSL-1 or triacylated Pam3CSK4 (102). In addition, NLRP7 is associated with recurrent miscarriages and recurrent hydatidiform molar pregnancies (100, 103–105). The above findings definitely support roles for NLRP2 and NLRP7 in inflammation and development. Interestingly, NLRP7 is not present in the mouse genome and appears to have arisen from a gene duplication event from NLRP2 (103). Therefore, it is not surprising that these two NLRs possess similar functions, but how they regulate both inflammasome activation and development is currently unknown (Table 1). Indeed, the role of NLRs in development is severely understudied, and many biochemical and cell specific studies on the function of these NLRs are needed to understand their differential roles. One possibility is that inflammasome activation is the mechanism by which NLRP2 and NLRP7 regulate embryonic development. The role of IL-1β in oocyte maturation and development has been appreciated for over a decade and has been reviewed previously (106, 107). Intrafollicular injection of IL-1β in horses induces ovulation but also inhibits embryo development (108), which is similar to the developmental arrest seen with NLRP2 and NLRP7 mutations. Furthermore, treatment of rabbit ovaries *in vitro* with IL-1β also arrests developing embryos (109). However, a lack of IL-1β signaling does not significantly affect fertility and embryo viability as IL-1 receptor deficient mice reproduce normally (110). Therefore, increased levels of IL-1β in patients with NLRP2 and NLRP7 mutations may be the cause of developmental arrest. However, much additional research on the roles of NLRP2 and NLRP7 needs to be performed before any conclusions can be reached regarding their functions in development.

## Conclusion

The role of NLRs in immune function is unequivocal. However, there is much molecular, biochemical and structural research which remains to be done to better understand how NLRs are activated and regulated. Due to the diversity of functions among NLRs, understanding their activation and regulation should provide a cornucopia of new opportunities to modulate the immune system. The activation of proinflammatory NLRs has already been demonstrated to be important for the function of many adjuvants used in research or in the clinic (111, 112). Targeting NLRs specifically for the generation of novel adjuvants may provide for more effective vaccines. On the other hand, targeting NLRs may provide for new treatments against numerous diseases such as arthritis (40, 113), diabetes (114, 115), colitis (44, 85, 116), multiple sclerosis (117–119), Alzheimer’s (49, 120), and many other diseases associated with mutations or disregulation of NLRs.

Several unstudied NLRs have recently been assigned some putative functions. NLRP10 has been reported to play a critical role in the induction of Th1 and Th17 mediated T cell responses through a defect in dendritic cells migration during *Candida albicans* infection (121, 122). As discussed above, NLRP7 was recently reported to assemble an inflammasome in response to bacterial diacylated lipopeptides (102). The fact that after a decade of research, new inflammasome activators are still being discovered may indicate that more NLRs fill this function than those previously described. Furthermore, recent studies have also validated roles for NLRP5 in embryonic development, although the exact mechanisms underlying these observations have not been elucidated (123–125). With more than 10 NLRs unstudied, it will be of interest to determine the function of these remaining NLRs in inflammation and development.

## Conflict of Interest Statement

The authors declare that the research was conducted in the absence of any commercial or financial relationships that could be construed as a potential conflict of interest.
